# The role and mechanism of bacterial outer membrane vesicles in the development of periodontitis

**DOI:** 10.3389/fmicb.2025.1654137

**Published:** 2025-09-03

**Authors:** Haocheng Wang, Jiale Sun, Guoxuan Ma, Fuping You, Boon Chin Heng, Yunyang Bai, Xuliang Deng

**Affiliations:** ^1^Beijing Key Laboratory of Tumor Systems Biology, NHC Key Laboratory of Medical Immunology, Department of Immunology, School of Basic Medical Sciences, Institute of Systems Biomedicine, Peking University Health Science Center, Beijing, China; ^2^Peking University School and Hospital of Stomatology, Beijing, China; ^3^Department of Dental Materials and Dental Medical Devices Testing Center, Peking University School and Hospital of Stomatology, Beijing, China; ^4^Department of Geriatric Dentistry, Peking University School and Hospital of Stomatology, Beijing, China

**Keywords:** outer membrane vesicles, periodontitis, biofilm, immunity, inflammation

## Abstract

Outer membrane vesicles (OMVs), nanoscale structures actively secreted by Gram-negative bacteria, have emerged as critical pathogenic components in periodontitis. While periodontitis has traditionally been associated with biofilm accumulation and bacterial colonization, recent studies highlight that OMVs contribute to disease progression independently of whole-cell bacterial presence. These vesicles are enriched with bioactive cargo such as lipopolysaccharides (LPS), proteases, DNA, and toxins, enabling them to persist in the periodontal microenvironment and interact with host immune and structural cells. They are also actively involved in biofilm formation and contribute to the development of antimicrobial resistance. Despite growing recognition of their involvement in periodontal disease, the extent of OMV interactions with host tissues and polymicrobial communities remains unclear. This review outlines the mechanisms through which OMVs influence inflammation, immune evasion, biofilm stability, and antibiotic resistance in periodontitis. It also highlights current knowledge gaps and concludes with potential therapeutic strategies targeting OMVs for the treatment of periodontitis.

## 1 Introduction

Periodontitis affects almost half of adults and more than 60% of those aged >65 years, while the Global Burden of Diseases 2017 study reported 796 million cases of severe periodontitis globally (Wu et al., 2022). A consensus has been reached that periodontitis is a microbial infection-driven inflammatory disease where biofilm composition significantly influences pathogenesis and tissue destruction (Sczepanik et al., 2020). While early models emphasized direct bacterial invasion or toxin secretion as primary mechanisms of host tissue damage, recent findings have uncovered a more sophisticated mode of host-pathogen communication: outer membrane vesicles (OMVs). These nanosized, spherical extracellular structures, typically ranging from 20 to 300 nm in diameter, are actively secreted by Gram-negative bacteria during growth. Composed of lipid bilayers, OMVs encapsulate a rich cargo of bioactive molecules derived from their parent bacteria. Through vesicle-mediated transfer, bacteria can deliver pathogenic molecules directly into host cells (Toyofuku et al., 2019).

In the context of periodontitis, OMVs serve as critical mediators that bridge bacterial biofilms within the subgingival environment and host periodontal tissue compartments. The small size and membrane fusion capability of OMVs enable them to penetrate biological membranes, matrices and epithelial barriers, delivering concentrated virulence factors deep into host tissues. Notably, OMVs derived from periodontal pathogens such as *Porphyromonas gingivalis* (*P. gingivalis*) can robustly stimulate the innate immune system. They activate macrophages, neutrophils, and other immune cells, inducing excessive production of pro-inflammatory cytokines.

Beyond immune activation, OMVs also modulate bone metabolism—a hallmark of periodontitis. Experimental studies have shown that OMVs from *P. gingivalis* and other oral pathogens can inhibit osteoblast differentiation and activity, while simultaneously promoting osteoclastogenesis and resorptive activity (Hienz et al., 2015). The dual destruction from inflammation and bone homeostasis imbalance accelerates the loss of supporting alveolar bone.

OMVs have emerged as key mediators in the pathogenesis of periodontitis. By delivering virulence factors, promoting local inflammation, contributing to antibiotic resistance, and facilitating immune evasion, OMVs play multiple roles in the disruption of periodontal homeostasis. This review aims to provide an overview of OMV biogenesis and structural characteristics, and to describe their involvement in inflammatory responses of periodontitis, antimicrobial resistance, and immune modulation. Finally, we discuss the potential of OMVs as novel diagnostic and therapeutic targets, offering new perspectives for the management of periodontal diseases.

## 2 Biogenesis, structure, and function of OMVs

Bacterial vesicles are nano-sized, spherical membrane-bound structures actively secreted by bacteria. These vesicles are typically enveloped by a lipid bilayer, which encapsulates various bioactive components derived from the parent cell (Rima et al., 2025). Both Gram-negative and Gram-positive bacteria are capable of producing such vesicles, though Gram-negative bacteria more efficiently generate them due to the presence of an outer membrane, resulting in structures referred to as OMVs. The biogenesis of bacterial vesicles in Gram-negative bacteria primarily occurs through two distinct mechanisms: outer membrane blebbing and explosive cell lysis.

Outer membrane blebbing occurs when localized separation takes place between the outer membrane and the peptidoglycan layer beneath it. This separation is often facilitated by the loss or redistribution of covalent linkages, such as those mediated by lipoproteins. When the outer membrane grows faster than the peptidoglycan scaffold, membrane blebs form, eventually budding off as OMVs (Roier et al., 2016). An alternative mechanism involves explosive lysis, in which membrane fragments generated during bacterial rupture reassemble into vesicular structures. For example, *Pseudomonas aeruginosa* (*P. aeruginosa*) predominantly produces vesicles via this lysis-driven process (Turnbull et al., 2016). This mechanism produces outer-inner membrane vesicles (OIMVs) and explosive outer membrane vesicles (EOMVs). Unlike OMVs formed through typical blebbing, EOMVs and OIMVs often contain cytoplasmic and inner membrane materials (Toyofuku et al., 2023).

The size of OMVs typically ranges between 20 and 300 nanometers, though this varies significantly between species (Mandelbaum et al., 2023). Because OMVs are derived from the parent cell’s outer membrane, their composition largely mirrors that of the outer membrane. Structurally, OMVs exhibit a phospholipid-rich inner leaflet and a lipopolysaccharide (LPS)-enriched outer leaflet, along with various outer membrane proteins and lipooligosaccharides (LOS) (Roier et al., 2016; Wood et al., 2025). Importantly, OMVs are not merely membrane fragments but also encapsulate a variety of bioactive molecules, including periplasmic components, peptidoglycan fragments, inner membrane and cytoplasmic proteins, nucleic acids (DNA and RNA), metabolites, metal ions, and signaling mediators (Zhao et al., 2025). The broad range of cargo highlights their functional versatility and biological importance.

Given that OMVs originate from Gram-negative bacteria and carry cargo highly similar to those of their parental bacteria, they can stimulate host cells in a manner similar to parental bacteria. Most OMVs are enriched in LPS, which serve as potent ligands for host pattern recognition receptors (PRRs), particularly Toll-like receptor 4 (TLR4). Therefore, LPS-bearing OMVs can activate inflammatory immune responses through TLR4-dependent signaling pathways involving both the MYD88 and TRIF adaptors (Ryu et al., 2017). In addition, the nucleic acid components contained within OMVs can engage host nucleic acid sensors, thereby triggering immune activation. For instance, OMVs derived from periodontal pathogens such as *P. gingivalis*, *Treponema denticola* (*T. denticola*), or *Tannerella forsythia* (*T. forsythia*) have been shown to activate the canonical inflammasome pathway in part through the cytosolic double-stranded DNA sensor AIM2. This activation leads to ASC/Caspase-1-dependent processing and secretion of bioactive IL-1β (Cecil et al., 2017). Owing to their membrane structure, OMVs can also deliver LPS into the cytosol of host macrophages via clathrin-mediated endocytosis. This intracellular delivery enables the activation of non-canonical inflammasome pathways, which depend on caspase-4 and -5 in humans, or caspase-11 in mice. Once activated, these caspases cleave gasdermin D, leading to its pore-forming activity and the induction of pyroptosis (Kayagaki et al., 2013; Vanaja et al., 2016; Santos et al., 2018). In addition to directly stimulating PRRs to trigger inflammation, OMVs can also promote macrophage apoptosis by activating pro-apoptotic molecules such as BAK. Moreover, OMV exposure has been shown to induce mitochondrial dysfunction, thereby initiating intrinsic mitochondrial apoptosis pathways (Deo et al., 2020).

Similar to the gut mucosa, the oral mucosa harbors a diverse and dense microbial community. However, unlike the single-layered epithelium of the gut, the oral epithelium consists of multiple stratified layers. This multilayered architecture alters how bacteria adhere to, interact with, and potentially penetrate the mucosal barrier, resulting in distinct colonization dynamics and barrier disruption mechanisms (Suárez et al., 2021). Moreover, the oral cavity comprises various anatomically and functionally distinct sites, including the buccal mucosa, keratinized gingiva, hard palate, throat, palatine tonsils, and tongue dorsum. These regions are differentially exposed to mechanical forces from mastication as well as microbial stimuli from food intake and respiration. As a result, each site harbors a unique microbial community shaped by its local environment and functional demands (Maki et al., 2021). Among the microbial communities implicated in periodontitis, key Gram-negative pathogens include *P. gingivalis*, *T. denticola*, *T. forsythia*, and *Fusobacterium nucleatum* (*F. nucleatum*). Notably, the oral mucosa lacks classical mucosa-associated lymphoid tissue (MALT), which underscores the critical role of antigen-presenting cells such as dendritic cells (DCs) in this context. These cells are thought to be essential for capturing bacterial antigens, migrating to oral lymphoid foci, and presenting antigens to T cells, thereby orchestrating adaptive immune responses during periodontal disease (Suárez et al., 2021).

OMVs play multifaceted roles in bacterial survival and pathogenesis. OMVs derived from various oral and opportunistic bacteria exhibit diverse pathogenic functions and colonization patterns within the oral cavity. *P. gingivalis* OMVs promote immune evasion, activate inflammasomes, and disrupt the epithelial barrier, with the bacterium predominantly colonizing the periodontal pocket (Cecil et al., 2017; Du Teil Espina et al., 2022; Katz et al., 2000, 2002; Takeuchi et al., 2019). Similarly, OMVs from *T. denticola* and *T. forsythia*, both localized to periodontal pockets, activate AIM2 and non-canonical inflammasomes, contributing to periodontal inflammation (Cecil et al., 2017). *F. nucleatum*, which resides in both subgingival and supragingival sites, releases OMVs that induce macrophage M1 polarization and enhance pro-inflammatory signaling (Chen J. et al., 2022; Chen G. et al., 2022). Opportunistically present in the oral cavity under dysbiotic conditions (Hernández-Jaimes et al., 2023), *Escherichia coli* (*E. coli*) secretes OMVs that stimulate dendritic cells to produce cytokines and release extracellular vesicles enriched in T cell-activating molecules (Diaz-Garrido et al., 2022). *Helicobacter pylori* (*H. pylori*), found in the gastric mucosa and oral cavity (Rodriguez-Archilla and Amhaouache, 2022), generates OMVs that promote M2 macrophage polarization, upregulate IL-10 expression, and suppress T cell responses, fostering immune tolerance (Ahmed et al., 2021). *Klebsiella pneumoniae* (*K. pneumoniae*), identified in periodontal pockets (Yamazaki and Kamada, 2024), produces OMVs capable of binding polymyxin B and disseminating resistance genes, thereby mediating cross-species antibiotic resistance (Burt et al., 2024; Tang et al., 2023). OMVs from Nontypeable *Haemophilus influenzae* (NTHi), which colonizes the nasopharynx and inflamed gingival sites, contribute to aberrant B cell activation and eventual exhaustion (Deknuydt et al., 2014). *Bacteroides* spp., commonly found in the oral cavity, secrete OMVs containing surface-associated β-lactamases and contribute to polymicrobial resistance (Stentz et al., 2015). OMVs promote antibiotic resistance (Stentz et al., 2015; Tang et al., 2023; Burt et al., 2024) and nutrient acquisition (Wang et al., 2020). OMVs mediate host-pathogen interactions by delivering virulence factors, modulating immune responses, and contributing to inflammation and immune evasion (Cecil et al., 2017; Wang et al., 2020; Diaz-Garrido et al., 2022; Du Teil Espina et al., 2022; Burt et al., 2024). These functional capacities highlight OMVs as dynamic mediators of microbial adaptation and periodontitis progression.

## 3 OMV-mediated pathogenesis in periodontitis

### 3.1 OMV-induced injury of non-immune cells

OMVs can exert direct cytotoxic effects on host cells. *P. gingivalis*-derived OMVs (*Pg* OMVs) have been shown to exert direct cytotoxic effects on various non-immune cells in periodontal tissue. For instance, *Pg* OMVs strongly suppress the proliferation of human periodontal ligament stem cells and downregulate osteogenic markers such as alkaline phosphatase (ALP), runt-related transcription factor 2 (RUNX2), and osterix (OSX), thereby impairing osteogenic differentiation (Mao et al., 2023). This suppression may contribute to alveolar bone loss commonly observed in periodontitis. Furthermore, *Pg* OMVs have been reported to induce caspase-3-dependent apoptosis in A549 epithelial cells, suggesting a potential mechanism by which similar epithelial cell damage may occur within the oral mucosa (He et al., 2020). Additionally, *Pg* OMVs can deliver microRNA-sized small RNAs (msRNAs), such as msRNA45033, into human periodontal ligament cells. This msRNA targets and suppresses chromobox protein homolog 5 (CBX5), a key component of heterochromatin involved in maintaining chromatin compaction and gene silencing, leading to hypomethylation of the TP53 promoter, increased p53 expression, and subsequent apoptosis (Fan et al., 2023). These findings collectively indicate that OMVs contribute to periodontal tissue destruction by directly impairing the viability and function of non-immune cells.

### 3.2 OMVs contribute to antibiotic resistance

OMVs have emerged as important mediators of antibiotic resistance. These vesicles enhance bacterial survival in the presence of antimicrobial agents through several distinct strategies, including antibiotic sequestration, enzymatic degradation, and horizontal gene transfer.

One key strategy involves OMVs acting as decoys that bind to antibiotics before they reach the bacterial cell surface, thereby reducing drug efficacy. For instance, *K. pneumoniae* secretes OMVs capable of binding polymyxin B, leading to increased resistance against this antibiotic. Notably, OMVs can also protect neighboring bacteria. *K. pneumoniae* OMVs have been shown to shield *E. coli*, *Salmonella* spp., *P. aeruginosa*, and *Legionella pneumophila* from polymyxin B-mediated killing, highlighting their role in cross-species protection within polymicrobial communities (Burt et al., 2024).

In addition to acting as decoys, OMVs can degrade antibiotics enzymatically. OMVs from *Bacteroides* spp., for example, contain surface-associated β-lactamases capable of hydrolyzing β-lactam antibiotics. These OMVs not only protect the producing bacteria but also extend protection to nearby commensal or pathogenic organisms, such as *Salmonella Typhimurium*, within polymicrobial environments like the gut (Stentz et al., 2015).

OMVs can also enhance antimicrobial resistance at the community level by mediating the transfer of resistance genes to recipient bacteria. For example, studies have shown that OMVs derived from carbapenem-resistant *K. pneumoniae* (CRKP) can carry the blaNDM-1 gene, which encodes a metallo-β-lactamase, and deliver it to other *K. pneumoniae* strains, including hypervirulent *K. pneumoniae* (hvKP). This horizontal gene transfer may contribute to the emergence and dissemination of carbapenem-resistant hypervirulent *K. pneumoniae* (CR-hvKP) (Tang et al., 2023).

Taken together, OMVs contribute to antibiotic resistance through multiple mechanisms. By sequestering antibiotics, degrading them enzymatically, and disseminating resistance genes, OMVs contribute to the resilience and adaptability of bacterial populations under antimicrobial pressure.

### 3.3 Immunomodulatory effects of OMVs on host immune cells

OMVs are not only vehicles for virulence factors and resistance determinants—they also interact with host immune cells, modulating immune responses. Multiple studies have shown that OMVs modulate the behavior of key host immune cells through various mechanisms, including neutrophils, macrophages, and DCs.

Neutrophils are among the first immune cells to respond during bacterial infection. OMVs can stimulate neutrophil activation and cytokine secretion. For example, OMVs from *Neisseria meningitidis* serogroup B induce polymorphonuclear neutrophils (PMNs) to release a range of pro-inflammatory cytokines and chemokines, including TNF-α, IL-1β, IL-8, MIP-1α, and MIP-1β (Lapinet et al., 2000). OMVs can also coat and disrupt host neutrophils. For example, OMVs from *P. gingivalis* can selectively coat the surface of neutrophils without being internalized. This targeted interaction induces degranulation, but notably, it does not trigger cell death (Du Teil Espina et al., 2022).

Macrophages are also key targets of OMVs. Upon interaction or endocytosis of OMVs, macrophages can produce large number of inflammatory mediators. OMVs derived from oral pathogens like *P. gingivalis*, *T. denticola*, and *F. nucleatum* have been shown to stimulate macrophages to secrete TNF-α, IL-1β, and IL-8 (Cecil et al., 2017). Some OMVs can even trigger programmed cell death. For instance, OMVs from *E. coli* activate caspase-1 in macrophages, leading to the cleavage of pro-IL-1β, pro-IL-18, and gasdermin D. This results in pyroptosis and the release of mature IL-1β and IL-18, amplifying the inflammation (Wang et al., 2020).

Importantly, OMVs also influence macrophage polarization. Macrophages exhibit phenotypic plasticity and can be polarized into either classically activated M1 (pro-inflammatory) or alternatively activated M2 (anti-inflammatory) phenotypes, depending on stimuli. Studies have shown that the OMVs of *F. nucleatum* can induce M0 macrophages to switch to the M1 phenotype, leading to the destruction of periodontal tissue (Chen J. et al., 2022; Chen G. et al., 2022). However, OMVs released by *H. pylori* SS1 can stimulate macrophages to polarize toward the M2 phenotype (Ahmed et al., 2021).

DCs, the primary antigen-presenting cells of the immune system, are also influenced by OMVs. Upon stimulation by OMVs, DCs upregulate surface molecules involved in antigen presentation and secrete cytokines that drive T cell activation. For instance, OMVs from probiotic and commensal strains of *E. coli* have been shown to stimulate DCs to release cytokines and to generate extracellular vesicles (EVs) enriched in molecules involved in T cell activation, thereby promoting robust T cell responses through both immune mediator secretion and direct cell–cell contact (Diaz-Garrido et al., 2022).

**Figure 1:**
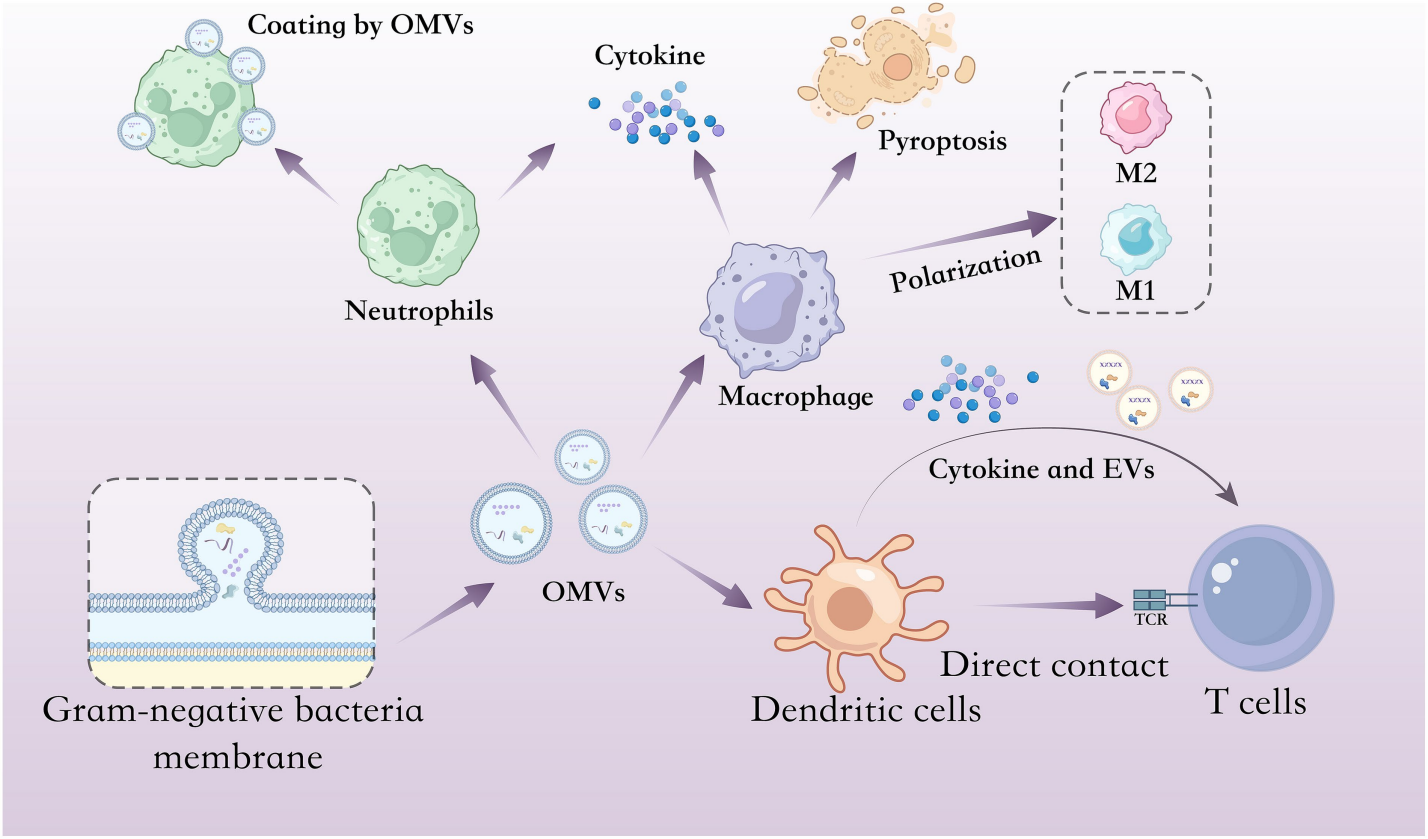
Interactions between OMVs and host immune cells. Schematic illustration of OMVs interacts with different host immune cells including neutrophils, macrophages, DCs and T cells. In neutrophils, OMVs can stimulate neutrophil activation and cytokine secretion but also coat and disrupt host neutrophils. In macrophages, OMVs can stimulate macrophages to produce cytokine and trigger pyroptosis; in addition, some OMVs can induce M0 macrophages to switch to either the M1 phenotype (pro-inflammatory) or the M2 phenotype (anti-inflammatory). In DCs and T cells, DCs stimulated by OMVs can upregulate surface molecules involved in antigen presentation, secrete cytokines that drive T cell activation and generate vesicles enriched in molecules involved in T cell activation, thereby promoting robust T cell responses through both immune mediator secretion and direct cell–cell contact.

In summary, OMVs are implicated in both antibiotic resistance and the modulation of host immune responses (Figure 1). By enhancing bacterial survival under selective pressures and actively shaping host–pathogen interactions, OMVs are critically involved in the pathogenesis and persistence of infections.

## 4 OMV-mediated immune evasion

In addition to directly causing inflammation, OMVs are pivotal to the chronic persistence of periodontitis by enabling immune evasion and supporting the establishment of drug-resistant biofilms. These functions help bacteria resist host clearance mechanisms and maintain long-term colonization.

One major immune evasion mechanism involves the ability of OMVs to act as decoys. These OMVs carry surface antigens similar to those of their parent bacteria, allowing them to bind host immune effectors such as antibodies, complement proteins, and antimicrobial peptides. By competing for binding with these immune effectors, OMVs damage the efficacy of immune factors and reduce the probability that the parent bacterial cells will be recognized or targeted. These decoys help shield parent bacteria from phagocytosis by macrophages and neutrophils. In this way, the immune system is distracted by vesicle-associated antigens, enabling bacterial persistence and immune evasion (Manning and Kuehn, 2011).

Another crucial immune evasion strategy of OMVs lies in their ability to directly disrupt host physical and humoral immune barriers through selectively enriched virulence factors. For instance, *Pg* OMVs are known to carry gingipains, which enzymatically degrade key transmembrane proteins involved in epithelial integrity, including tight junction proteins such as E-cadherin and occludin, as well as adhesion molecules like JAM1 (Katz et al., 2000, 2002; Takeuchi et al., 2019). In addition to proteolytic degradation, OMV-associated LPS can induce the production of pro-inflammatory mediators such as TNF-α and ROS, which further downregulate E-cadherin expression (Fujita et al., 2012; Abe-Yutori et al., 2017; Oh et al., 2019). Moreover, *Pg* OMVs may suppress the transcription factor GRHL2, a key regulator of epithelial junctional stability (Chen et al., 2019). These coordinated actions weaken the gingival epithelial barrier and facilitate bacterial penetration into underlying connective tissues.

OMVs can also directly modulate host immune cell function. For example, *Pg* OMVs downregulate CD14, a co-receptor critical for the detection of LPS, on macrophage surfaces. This suppression dampens the macrophages’ ability to produce effective innate immune responses (Duncan et al., 2004). Additionally, some OMVs induce immune suppression, shifting the host immune response from immune activation to immune tolerance. OMVs from *H. pylori* have been shown to induce IL-10 production in peripheral blood mononuclear cells, thereby suppressing CD4 + T cell proliferation and promoting T cell apoptosis. This creates a localized immunosuppressive microenvironment that favors bacterial survival (Winter et al., 2014). Other studies show that OMVs from NTHi can abnormally activate B cells through dual signaling via the IgD B cell receptor (BCR) and TLR9, rendering them hypersensitive to BAFF. This leads to excessive consumption of B cell resources and reduces the clonal expansion of antigen-specific B cells (Deknuydt et al., 2014).

Beyond immune interference, OMVs are critically involved in the formation and maintenance of biofilms on dental surfaces—an essential immune evasion mechanism that significantly contributes to the chronicity and antimicrobial resistance of periodontal infections. During early biofilm formation, extracellular DNA (eDNA) is critical for bacterial adhesion, and OMVs serve as a major source of eDNA. eDNA facilitates bacterial aggregation and biofilm formation on various material surfaces (Grande et al., 2015).

Meanwhile, OMVs also serve as structural units of biofilms and are key materials for constructing and stabilizing the solid structure of biofilms. Functionally, OMVs are integral components of the extracellular polymeric substance (EPS) matrix that stabilizes the biofilm. They contain and release proteins, polysaccharides, lipids, and nucleic acids—key macromolecules for EPS assembly. For example, OMVs derived from *H. pylori* can directly incorporate into the EPS matrix on polystyrene surfaces, mediated by a specific 22-kDa protein (Wang et al., 2015). Upon vesicle rupture or degradation, their cargo is incorporated into the surrounding biofilm environment, enhancing matrix density and cohesion. This matrix acts as a protective barrier, shielding the colonized bacterial community from antibiotics and immune effectors such as phagocytes, complement proteins, and antibodies.

Taken together, OMVs construct a complicated immune evasion mechanism by decoying host immunity, reprogramming immune responses, and reinforcing the structural and functional integrity of biofilms. These multiple roles help explain the therapeutic resistance and frequent recurrence of periodontitis. Consequently, targeting OMVs represents a promising strategy to disrupt bacterial defenses.

## 5 Conclusions and perspectives

OMVs play critical roles in the pathogenesis of periodontitis. These nanoscale vesicles are enriched in bioactive molecules including LPS, outer membrane proteins, toxins, enzymes, and nucleic acids. Once released into the periodontal microenvironment, OMVs contribute to disease development through multiple interrelated mechanisms.

Notably, they act as powerful immunostimulatory agents by delivering LPS and other pathogen-associated molecular patterns (PAMPs) directly to immune cells. This triggers the activation of macrophages, neutrophils and DCs, leading to the release of key pro-inflammatory cytokines such as TNF-α, IL-1β, and IL-8. The resulting inflammatory cascade contributes to periodontal tissue destruction. Furthermore, OMVs influence immune cell behavior, including the polarization of macrophages and the maturation of DCs, ultimately driving immune dysregulation and sustaining chronic inflammation. At the same time, OMVs help bacteria evade host immune defenses. By displaying surface antigens that resemble those of the parent bacterium, OMVs can bind host antibodies and complement proteins, effectively distracting these immune effectors from targeting viable pathogens. This decoy function allows bacteria to persist in the periodontal environment without being cleared. OMVs also interfere with immune recognition by downregulating key pattern recognition receptors such as CD14 on macrophages, thereby suppressing innate immune activation and allowing bacterial colonization to continue unchallenged.

OMVs are actively involved in the formation and stabilization of biofilms. They facilitate initial bacterial adhesion by serving as adhesive units and sources of eDNA. OMVs also provide structural materials, such as polysaccharides, proteins, and lipids, that enhance the density and stability of the EPS surrounding biofilm communities. This biofilm structure protects bacteria from antibiotics and immune clearance, contributing to the chronicity and recurrence of periodontitis. Furthermore, OMVs contribute to antimicrobial resistance not only by shielding bacteria from antibiotics but also by delivering β-lactamase enzymes and transferring resistance genes, such as blaNDM-1, to nearby bacteria. These functions amplify the resilience of bacterial communities against antimicrobial therapies.

Collectively, OMVs serve as multifunctional pathogenic mediators in periodontitis, promoting inflammation, immune evasion, biofilm maturation, and antibiotic resistance. Their stability in the periodontal microenvironment, high abundance, and capacity to carry diverse virulence factors make them a driving force behind the persistence and progression of periodontal disease.

Despite increasing recognition of OMVs in periodontitis, critical gaps remain in understanding their interactions with both the host and other microbes. Most current studies have focused on the effects of OMVs derived from a single pathogen, such as *P. gingivalis*, on isolated host immune responses. However, periodontitis occurs within a polymicrobial context where interspecies communication and cooperation shape disease progression and clinical outcomes.

It remains unclear how OMVs influence other bacteria within biofilm communities. For example, OMVs may promote the horizontal transfer of virulence or resistance traits to neighboring species, or alter the local microenvironment to favor pathogenic over commensal species. Furthermore, how OMVs interact with epithelial cells, fibroblasts, or osteoblasts in periodontal tissues has yet to be fully elucidated. These interactions may significantly affect tissue remodeling, immune signaling, or bacterial colonization dynamics. A comprehensive understanding of OMV-mediated microbial interactions is fundamental to defining their role in periodontal homeostasis and dysbiosis. Future research is expected to explore how OMVs modulate microbial crosstalk and manipulate host barrier functions to promote chronic inflammation and disease persistence.

Given their central involvement in driving inflammation, immune evasion, and biofilm maturation, OMVs represent a compelling therapeutic target in periodontitis. Unlike whole bacteria, OMVs are acellular, structurally stable, and persist in the periodontal microenvironment even after bacterial loads are reduced by antibiotics. This residual OMV activity continues to promote tissue destruction and chronic inflammation, contributing to disease recurrence.

Therapeutic strategies aimed at neutralizing OMVs could involve the use of engineered molecules or natural compounds that bind and inactivate vesicular cargo—such as LPS, proteases, and adhesins—thereby blocking the delivery of virulence factors. For instance, curcumin has been shown to attenuate OMV-induced cytotoxicity, while endotoxin-binding agents like polymyxin derivatives may sequester LPS (Izui et al., 2021). Additionally, cannabidiol (CBD) has been identified as a potent modulator of bacterial membrane vesicles. CBD significantly inhibits the release of OMVs from Gram-negative bacteria, such as *E. coli*, by disrupting the biogenesis of these vesicles, which could potentially reduce their role in antibiotic resistance and biofilm formation (Kosgodage et al., 2019a). Furthermore, studies have indicated that peptidylarginine deiminase (PAD) inhibitors can also be utilized to reduce OMV release from Gram-negative bacteria. These PAD inhibitors, such as Cl-amidine and BB-Cl-amidine, have been shown to reduce the release of OMVs from *E. coli*, subsequently enhancing the efficacy of antibiotics (Kosgodage et al., 2019b). In addition to inhibiting OMV production, blocking the pathogenic effects of OMV cargo is also key to targeting OMV-based therapies. The selective inhibition of LPS/TLR4 signaling by TAK-242 is one such promising therapeutic strategy. TAK-242 has been shown to block LPS-induced inflammation by specifically targeting the intracellular domain of TLR4, thus reducing the excessive inflammatory response that contributes to conditions like sepsis (Kawamoto et al., 2008).

Importantly, OMV-targeted interventions may complement conventional antimicrobial or host-directed therapies by disrupting key virulence pathways without imposing additional selective pressure for resistance. Altogether, targeting OMVs offers a promising direction for the development of adjunctive treatments aimed at mitigating the persistent inflammatory and immunomodulatory effects of OMVs. More research needs to focus on identifying OMV-specific molecular targets, optimizing delivery platforms, and evaluating the efficacy of OMV-blocking agents in preclinical models of periodontitis.
